# Prevalence of abnormal glucose values and gestational diabetes mellitus among pregnant women in Xi’an from 2015 to 2021

**DOI:** 10.1186/s12884-023-05798-w

**Published:** 2023-06-24

**Authors:** Gai Li Meng, Qi Wang, Ru Kang, Xiao Yue Cheng, Jun Lan Yang, Yun Xie

**Affiliations:** 1 Department of Clinical Laboratory, Northwest Women’s and Children’s Hospital, Xi’an, 710061 China; 2 Department of Clinical Laboratory, Second Affiliated Hospital of Xi’an Jiaotong University, Xi’an, 710061 China

**Keywords:** Gestational diabetes mellitus, Oral glucose tolerance test, Xi’an

## Abstract

**Background:**

Pregnant women with gestational diabetes mellitus (GDM) often have an increased risk of adverse pregnancy outcomes. The purpose of this study was to explore the prevalence and characteristics of GDM in Xi’an from 2015 to 2021 since the implementation of China’s “Two-Child policy” and to provide a clinical basis for the management of GDM.

**Methods:**

We analyzed the oral glucose tolerance test (OGTT) results of 152,836 pregnant women who underwent routine prenatal examination at the Northwest Women and Children’s Hospital from 2015 to 2021. Additionally, we analyzed the GDM prevalence and characteristics.

**Results:**

The prevalence of GDM in the Xi’an urban area was 24.66% and exhibited an increasing trend annually (χ2 for trend = 43.922, p < 0.001) and with age (χ2 for trend = 2527.000, p < 0.001). Consistent with this, the proportion of pregnant women aged 18–25 and 26–30 years decreased significantly with the annual growth (χ2 for trend = 183.279, p < 0.001 and χ2 for trend = 33.192, p < 0.001, respectively). The proportion of pregnant women aged 31–35 and 36–42 years increased gradually annually (χ2 for trend = 134.436, p < 0.001and χ2 for trend = 44.403, p < 0.001, respectively). Of the pregnant women diagnosed with GDM, 71.15% (65.05–74.95%) had abnormal fasting plasma glucose (FPG) values. The highest percentage of patients had a single abnormal OGTT value (68.31%; 65.77–70.61%), followed by two (20.52%; 18.79–22.55%) and three (11.17%; 10.11–11.85%) abnormal values (FPG and 1-h and 2-h plasma glucose (PG).

**Conclusion:**

The prevalence of GDM among pregnant women in Xi’an region was high, and it had a increasing trend over the period from 2015 to 2021. Notably, the proportion of elder pregnant women, aged 31–42 years, presented a significant rise after the implementation of the universal two-child policy. On the basis of the high incidence of GDM among elder pregnant women and the high rate of abnormal OGTT values (numbe ≥ 2) in pregnant women diagnosed with GDM, the management of GDM should be intensified, and relevant departments should pay more attention to pregnant women of advanced age.

## Background

Gestation diabetes mellitus (GDM) traditionally refers to the first occurrence or discovery of abnormal glucose metabolism during pregnancy. Pregnancy is the strongest risk factor for GDM because of the significant physiological changes in blood glucose regulation during pregnancy. Due to the substantial increase in the levels of growth hormone, adrenocorticotropic hormone, human placental prolactin, pituitary prolactin, estrogen, and progesterone in the systemic circulation, insulin resistance gradually increases and typically manifests during the second and third trimesters of pregnancy [1]. The pathogenesis and characteristics of GDM are insulin resistance and relative insulin deficiency caused by decreased cell function and quality [2, 3]. With the continuous improvement of people’s standard of living, GDM has become the most common complication during pregnancy [4].

GDM has long been associated with obstetric and neonatal complications. Pregnancies complicated by GDM are characterized by important changes in the placental expression of neoangiogenesis and inflammation markers which may be associated with adverse perinatal outcomes [5, 6]. Maternal hyperglycemia promotes accelerated fetal growth, leading to macrosomia, large for gestational age infant, and increased risk of cesarean section and delivery trauma, including perineal laceration, uterine rupture, shoulder dystocia, brachial plexus injury, fracture, and perinatal asphyxia [7]. Also, GDM increases the risk of neonatal metabolic abnormalities, such as neonatal respiratory distress syndrome, polycythemia, hyperbilirubinemia, neonatal hypoglycemia, and hyperinsulinemia [8]. It is an independent risk factor for type 2 diabetes, obesity, hypertension, and metabolic syndrome in postpartum women and their offspring [9]. Furthermore,GDM is increasingly recognized as a risk factor for future maternal and offspring cardiometabolic disease [10]. Therefore, it is essential to detect and diagnose GDM as early as possible by monitoring the blood glucose level during pregnancy, especially during the early pregnancy, and to treat and control the blood glucose levels in a timely manner.

The prevalence of GDM varies widely, depending on the population. East Asians, Hispanics, blacks, and Indians have high risk of GDM [11]. In addition, increased maternal age is a risk factor for GDM [12]. Previous studies demonstrated a positive relationship between increasing maternal age and risk for GDM [12–15]. Although the prevalence of GDM has been reported in some regions in China, the average prevalence rate of GDM and its epidemic characteristics (the changes of maternal age after the implementation of the universal two-child policy) in Xi’an (Northwest China) have not been explored.

In this study, we investigated the prevalence of GDM, and the characteristics of maternal age and abnormal glucose values among pregnant women diagnosed with GDM in Northwest Women’s and Children’s Hospital from 2015 to 2021, to provide a clinical basis for GDM management in Xi’an.

## Methods

### Participants and study design

This was a retrospective study conducted at Northwest women’s and children’s hospital, a tertiary maternity and pediatric hospital, with 20,000 births annually.

The study was reviewed and approved by the Ethics Committee of the Northwest Women’s and Children’s hospital (approval No. 21–16). Individual consent for this retrospective study was waived by the Ethics Committee of the Northwest Women’s and Children’s hospital. Data related to the OGTT were obtained from the electronic medical records of 152,836 cases between 1 January 2015 and 31 December 2021. The exclusion criteria included participants younger than 18 years, pre-gestational diabetes mellitus (PGDM), the overt diabetes mellitus (DM) of FPG > 7.0 mmol/L(the fasting plasma glucose test was carried out during the first trimester of gestation) [16], duplicate records, a missing diagnosis of GDM, and non-resident persons who visit our hospital. The ages of the pregnant women ranged from 18 to 42 years, with an average age of 29 ± 2.38 years.

### OGTT specimen collection and processing

All participants who underwent OGTT fasted for at least 8 h before blood collection. After fasting venous blood collection, the women were requested to drink 75 g of anhydrous glucose dissolved in 250 mL of water within 5 min. Venous blood was collected after 1 and 2 h of oral glucose water ingestion. The venous blood was collected in citric acid sodium fluoride anticoagulation tubes (BD Biosciences, USA). Centrifugation at 1,000×g for 10 min was performed within 1 h of sample collection to separate the plasma.

### Plasma glucose level detection

Beckman Coulter AU5800 automatic biochemical analyzer (USA) was used. The glucose detection reagents and calibrators were produced by Beckman Coulter (USA). The biochemical composite quality control products (BIO-RAD company) were used as the quality control products, including for low and high levels. All tests were performed in strict accordance with the operating procedures of the instruments and reagents.

### Diagnostic criteria of GDM according to the International Association of Diabetes and Dignity Study Group (IADPSG)

A 75-g OGTT was performed at 24–28 weeks of pregnancy. The normal values at fasting and 1-h and 2-h plasma glucose (PG) were 5.1, 10.0, and 8.5 mmol/L, respectively. GDM was diagnosed if any of the values reached or exceeded these values.

### Statistical methods

SPSS software (version 18.0; IBM Corp., Armonk, NY, USA) was used for data analysis. Numerical data were expressed as the number of cases (n) and percentage (%). Tests for difference were two-sided. Changes in the trend were analyzed using the Cochran Armitage trend test. The test level was α = 0.05. P < 0.05 was considered to indicate a statistically significant difference.

## Results

### Distribution of GDM prevalence in different years

As shown in the Table 1, from 2015 to 2021, the prevalence of GDM in Xi’an was as high as 24.66%. The lowest prevalence was in 2017 (21.36%) and the highest was in 2019 (26.91%). There were significant differences in the prevalence of GDM in different years (χ^2^ = 254.200, *p* < 0.001). Analysis of the prevalence of GDM according to the years showed a slight increasing trend over time (χ ^2^ for trend = 43.922, *p* < 0.001).

**Table 1:** Distribution of Gestation diabetes mellitus (GDM) prevalence in different years [n (%)]

Year	2015	2016	2017	2018	2019	2020	2021	Total
**GDM (n)**	3,273	5,563	4,736	5,605	7,190	5,387	5,940	37,694
**Cases (n)**	13,597	21,874	22,176	23,626	26,723	22,114	22,726	152,836
**GDM prevalence (%)**	24.07	25.43	21.36	23.72	26.91	24.36	26.14	24.66

### Distribution of GDM prevalence according to age

The study divided pregnant women with GDM into four age groups: 18–25, 26–30, 31–35, and 36–42 years. Table 2 showed that the prevalence of GDM was 16.38%, 21.59%, 28.62%, and 37.40% in the aforementioned age groups, respectively. There was a significant difference in the GDM prevalence between the age groups (χ^2^ = 2568.000, *p* < 0.001). The prevalence of GDM showed an increasing trend with age (χ^2^ for trend = 2527.000, *p* < 0.001) from 2015 to 2021. Additionally, the prevalence of GDM among pregnant women aged 18–25 years was low (12.13–19.25%), which was slightly lower than that for women aged 26–30 years (17.81–23.79%). This was in turn significantly lower than the prevalence of GDM in women aged 31–35 years (from 25.45 to 30.42%) and was as high as 34.74–41.40% in women aged 36–42 years.

**Table 2:** Distribution of GDM prevalence according to age groups [n (%)]

Age (years)	2015	2016	2017	2018	2019	2020	2021	Total
**18–25**	15.44	16.89	12.13	14.92	19.32	17.08	19.25	16.38
	(326/2112)	(398/2357)	(274/2259)	(361/2420)	(498/2578)	(305/1786)	(346/1797)	(2508/15,309)
**26–30**	22.31	22.62	17.81	20.99	23.79	20.97	22.33	21.59
	(1585/7104)	(2627/11,612)	(1957/10,986)	(2502/11,918)	(3263/13,716)	(2243/10,698)	(2408/10,783)	(16,585/76,817)
**31–35**	29.98	30.27	25.45	27.57	30.42	27.38	29.67	28.62
	(1008/3362)	(1797/5936)	(1648/6475)	(1953/7085)	(2462/8093)	(2094/7649)	(2418/8150)	(13,380/46,750)
**36–42**	34.74	37.63	34.89	35.81	41.40	37.61	38.48	37.40
	(354/1019)	(741/1969)	(857/2456)	(789/2203)	(967/2336)	(745/1981)	(768/1996)	(5221/13,960)
**χ** ^**2**^ **for trend** **/** ***P***	**223.862/0.000**	**364.633/0.000**	**521.478/0.000**	**386.050/0.000**	**422.022/0.000**	**327.569/0.000**	**320.194/0.000**	**2527.000/0.000**
**χ** ^**2**^ **/** ***P***	**225.900/0.000**	**367.000/0.000**	**529.300/0.000**	**388.600/0.000**	**443.400/0.000**	**344.700/0.000**	**335.100/0.000**	**2568.000/0.000**

### Proportion of pregnant women with GDM according to age and year

As shown in the Table 3, from 2015 to 2021, the proportion of pregnant women aged 18–25 years in Xi’an decreased significantly, from 15.53% in 2015 to 7.91% in 2021 (χ^2^ for trend = 183.279, *p* < 0.001). The proportion of pregnant women with GDM aged 26–30 years also gradually decreased over the years, from 52.25% in 2015 to 47.45% in 2021 (χ^2^ for trend = 33.192, *p* < 0.001). The proportion of pregnant women with GDM aged 31–35 years increased gradually over the years, from 24.73% in 2015 to 35.96% in 2021 (χ^2^ for trend = 134.436, *p* < 0.001). The proportion of pregnant women with GDM aged 36–42 years also gradually increased over the years, from 7.49% in 2015 to 8.78% in 2021 (χ^2^ for trend = 44.403, *p* < 0.001).

**Table 3:** Composition of pregnant women at different ages [n (%)]

Age (years)	2015	2016	2017	2018	2019	2020	2021	χ^2^ /*P*	χ^2^ for trend /*P*
**18 – 25**	15.53	10.78	10.19	10.24	9.65	8.08	7.91	**303.7**	**183.279**
	(2112/13,597)	(2357/21,874)	(2259/22,176)	(2420/23,626)	(2578/26,723)	(1786/22,114)	(1797/22,726)	**0.000**	**0.000**
**26 – 30**	52.25	53.09	49.54	50.44	51.33	48.38	47.45	**66.951**	**33.192**
	(7104/13,597)	(11,612/21,874)	(10,986/22,176)	(11,918/23,626)	(13,716/26,723)	(10,698/22,114)	(10,783/22,726)	**0.000**	**0.000**
**31– 35**	24.73	27.14	29.20	29.99	30.28	34.59	35.86	**141.5**	**134.436**
	(3362/13,597)	(5936/21,874)	(6475/22,176)	(7085/23,626)	(8093/26,723)	(7649/22,114)	(8150/22,726)	**0.000**	**0.000**
**36– 42**	7.49	9.00	11.08	9.32	8.74	8.96	8.78	**135.2**	**44.403**
	(1019/13,597)	(1969/21,874)	(2456/22,176)	(2203/23,626)	(2336/26,723)	(1981/22,114)	(1996/22,726)	**0.000**	**0.000**

### Composition of different GDM types

This study analyzed the results of GDM diagnosed by OGTT from 2015 to 2021. As shown in the Table 4, although the proportion of abnormal FPG values was 71.15% (65.05–74.95%), if the 1-hPG and 2-hPG were excluded, the missed diagnosis rate would be as high as 28.85% (25.05–34.95%). The number of abnormal OGTT values showed that pregnant women with GDM in this region had one, two, and three abnormal values in 68.31% (65.77–70.61%), 20.52% (18.79–22.55%), and 11.17% (10.11–11.85%) of cases, respectively. Of them, the highest proportion of patients had a single abnormal FPG value in OGTT (46.66%; 40.10–51.79%), followed by a single abnormal 2-hPG value (11.94%; 10.04–14.61%) and a single abnormal 1hPG value (9.72%; 7.97–11.06%). Of the two OGTT numerical abnormalities, FPG and 1hPG accounted for the highest abnormal values (8.06%; 7.08–9.24%), followed by 1-hPG and 2-hPG abnormal values (7.19%; 5.88–9.27%) and FPG and 2-hPG abnormal values (5.27%; 4.80–5.68%).

**Table 4:** Composition of GDM types [n (%)]

GDM types			2015	2016	2017	2018	2019	2020	2021	Total	χ^2^ /*P*	χ^2^ trend /*P*
FPG	1hPG	2hPG										
**+**	**-**	**-**	51.79	46.54	40.10	43.94	50.07	48.45	45.98	46.66	**174.80**	0.556
			(1695/3273)	(2589/5563)	(1899/4736)	(2463/5605)	(3600/7190)	(2610/5387)	(2731/5940)	(17,587/37,694)	**0.000**	0.456
**+**	**-**	**+**	5.68	5.57	5.22	4.80	5.08	5.36	5.39	5.27	5.454	0.435
			(186/3273)	(310/5563)	(247/4736)	(269/5605)	(365/7190)	(289/5387)	(320/5940)	(1986/37,694)	0.487	0.510
**+**	**+**	**-**	7.36	7.08	8.07	9.24	7.83	8.98	7.66	8.06	**27.899**	**3.547**
			(241/3273)	(394/5563)	(382/4736)	(518/5605)	(563/7190)	(484/5387)	(455/5940)	(3037/37,694)	**0.000**	**0.060**
**+**	**+**	**+**	10.11	10.70	11.68	11.42	11.00	11.06	11.85	11.17	9.571	**3.943**
			(331/3273)	(595/5563)	(553/4736)	(640/5605)	(791/7190)	(596/5387)	(704/5940)	(4210/37,694)	0.144	**0.047**
**-**	**-**	**+**	10.85	13.41	14.61	12.44	11.53	10.04	10.77	11.94	**75.890**	**27.256**
			(355/3273)	(746/5563)	(692/4736)	(697/5605)	(829/7190)	(541/5387)	(640/5940)	(4500/37,694)	**0.000**	**0.000**
**-**	**+**	**-**	7.97	9.20	11.06	10.53	8.61	9.95	10.45	9.72	**41.056**	**4.879**
			(261/3273)	(512/5563)	(524/4736)	(590/5605)	(619/7190)	(536/5387)	(621/5940)	(3663/37,694)	**0.000**	**0.027**
**-**	**+**	**+**	6.23	7.50	9.27	7.64	5.88	6.14	7.90	7.19	**69.273**	1.577
			(204/3273)	(417/5563)	(439/4736)	(428/5605)	(423/7190)	(331/5387)	(469/5940)	(2711/37,694)	**0.000**	0.209

## Discussion

GDM is a common complication of pregnancy. According to the data from the International Diabetes Federation published in 2017, the prevalence of GDM is almost 14% globally, 9% in Africa, 12.3% in North America, and 21% in Asia [17]. In China, the prevalence of GDM varies in different regions. Using the IADPSG standard, some studies have found that the prevalence of GDM was as high as 24.24% in Beijing [18], 21.8% in Qingdao, Shandong Province [19], 22.94% in Guangzhou, Guangdong Province [20] and 18.3% in Chengdu, Sichuan Province [21]. The differences in the prevalence of GDM in different regions of China may be related to differences in geographic locations, nationalities, and dietary and lifestyle habits; however, the exact reasons are unclear. The present study shows that, from 2015 to 2021, the prevalence rate of GDM in Xi’an city was as high as 24.66%, which is significantly higher than that reported previously from China. Therefore, more attention should be paid to the glucose metabolism of pregnant women in this region. This study found that the prevalence of GDM in Xi’an City showed a slight increasing trend over the years from 2015 to 2021, which was consistent with recent study results from around the world [17, 18, 20].

Maternal age is an important risk factor for GDM. Some studies showed that the risk of GDM increases by 12.74% per annum when a pregnant woman is over the age of 18 years [12–14]. Another study showed that pregnant women aged 36–45 years have a 4-fold higher risk of GDM than those aged 18–25 years [15]. This study showed that the prevalence of GDM among pregnant women in Xi’an city was 16.38%, 21.59%, 28.62%, and 37.40% in women aged 18–25, 26–30, 31–35, and 36–42 years, respectively. The prevalence rate of GDM among pregnant women aged 36–42 years was about twice that of those aged 18–25 years and showed an increasing trend with increasing age, which could be the important factor for increasing trend over the period between 2015 and 2021. Therefore, it is essential to provide health education related to GDM for older women during pre-pregnancy, pregnancy, and postpartum stages to reduce the risk of GDM and its adverse pregnancy outcomes.

To analyze the changing trends of the proportion of pregnant women in different age groups over the years in Xi’an, the pregnant women were divided into those aged 18–25, 26–30, 31–35, and 36–42 years. The results showed that the proportion of pregnant women in the two youngest age groups of 18–25 and 26–30 years in Xi’an decreased from 67.78% to 2015 to 55.36% in 2021. However, the proportion of pregnant women with GDM in the older age groups of 31–35 and 36–42 years increased from 32.22% to 2015 to 44.64% in 2021. Since 2015, China has implemented the “China two-child policy”, which may be the main reasons for this changing trend. And this changing trend may be the main reason for the increasing incidence of GDM. Therefore, the prevention, screening, control, and treatment of GDM and the reduction of adverse pregnancy outcomes would be significant challenges in the future.

Some studies have found that abnormal FPG values are positively related to the increased risk of adverse pregnancy outcomes, such as spontaneous abortion, premature delivery, macrosomia, small for gestational age, and perinatal death. Every 1 mmol/L increase in the FPG level is associated with increased risks of spontaneous abortion, premature delivery, macrosomia, small for gestational age, and perinatal death by 8%, 3%, 5%, 3%, and 9%, respectively [22]. This study summarized and analyzed the OGTT results and found that 71.15% (65.05–74.95%) of pregnant women diagnosed with GDM in this region had abnormal FPG values. Screening fasting blood glucose levels before pregnancy and during early pregnancy, as well as taking effective measures to control the fasting blood glucose levels, should be the focus of eugenics in this region.

High OGTT values often indicate serious glucose metabolism disorders and insulin resistance. The number of abnormal OGTT values is related to the overall incidence of adverse pregnancy outcomes, such as cesarean section, premature delivery, pregnancy-induced hypertension, premature rupture of membranes, preeclampsia, macrosomia, neonatal asphyxia, and full-term low birth weight infants [23]. This study analyzed the number of abnormal OGTT values in pregnant women diagnosed with GDM and found that one, two, and three abnormal OGTT values were present in 68.31% (65.77–70.61%), 20.52% (18.79–22.55%), and 11.17% (10.11–11.85%) of patients, respectively. Although the proportion of GDM pregnant women with three abnormal values in OGTT was the lowest, it was still higher than 10%. Considering the high prevalence of GDM in this region and the large population base, it is essential to advise the appropriate diet, exercise, and insulin to strictly control the blood glucose levels of pregnant women and reduce the incidence of adverse pregnancy outcomes.

Although the current‘gold standard’for GDM diagnosis is still the OGTT, the pre-analytical and physiological factors, as well as analytical and post-analytical limitations have been reported for the OGTT [10, 24]. Recently new studies demonstrated even women with normal OGTT show abnormal glycemic fluctuations, based on this point, a new method called CGM (Continuous Glucose Monitoring) to detect dysglycemia in pregnancy, might help to detect glycemic fluctuations in women with normal OGTT, improving their treatment and outcomes [25, 26]. Further, various studies already have proposed prediction models that may help to detect women at risk of GDM in the first trimester using ultrasound or biochemical markers [27–31], which may reduce the risk of adverse pregnancy outcomes.

Our study had several strengths. First, 152,836 pregnant women were included in this study, which is a large sample size. Also, the data was collected from 2015 to 2021 since the first year of the implementation of the comprehensive two-child policy. Nevertheless, our study had certain limitations. We only analyzed data from the 75-g OGTT performed at 24–28 weeks of gestation. Additionally, we did not collect participants’ information regarding the use of hypoglycemic drugs before and during pregnancy or other characteristics. Therefore, our results may underestimate the risk of GDM. Furthermore, this is a single-center retrospective study, which may limit its external validity.

## Conclusion

In conclusion, the prevalence of GDM among pregnant women in Xi’an region was high, and it had a increasing trend over the period from 2015 to 2021. Notably, the proportion of elder pregnant women, aged 31–42 years, presented a significant rise after the implementation of the universal two-child policy. On the basis of the high incidence of GDM among elder pregnant women and the high rate of abnormal OGTT values (number ≥ 2) in pregnant women diagnosed with GDM, the management of GDM should be intensified, and relevant departments should pay more attention to pregnant women of advanced age. In the future, it is urgent to set a special study on the causes of the increasing prevalence of GDM in Xi’an and the long-term impact of the two-child policy on GDM.

## Data Availability

The detailed datasets used and analyzed during the current study availiable from the corresponding and first authors.
